# Arterial supply of the interpeduncular part of the human oculomotor nerve

**DOI:** 10.3389/fnana.2026.1758626

**Published:** 2026-03-03

**Authors:** Samra Hajrović, Ema Bexheti, Aleksandra Dožić, Sadi Bexheti, Dejan Ćetković, Helena Marić Kujundžić, Marko Simić, Sonja Milašinović, Uroš Mirčić, Zdravko Vitošević, Milan Milisavljević, Aleksandar Mirčić, Mila Ćetković

**Affiliations:** 1 Ophthalmology Division, General Hospital, Novi Pazar, Serbia; 2 Faculty of Medical Science, Institute of Anatomy, State University of Tetova, Tetova, North Macedonia; 3 Laboratory for Vascular Morphology, Faculty of Dental Medicine, Institute of Anatomy, University of Belgrade, Belgrade, Serbia; 4 Faculty of Medicine Foča, University of East Sarajevo, Foča, Bosnia and Herzegovina; 5 Clinic for Orthopedic Surgery and Traumatology, University Clinical Center of Serbia, Belgrade, Serbia; 6 Institute for Children’s Disease, Clinical Centre of Montenegro, Podgorica, Montenegro; 7 Centre for Radiology, University Clinical Centre of Serbia, Belgrade, Serbia; 8 Department of Anatomy, Faculty of Medicine, University in Priština – Kosovska Mitrovica, Kosovska Mitrovica, Serbia; 9 Academy of Medical Sciences, Serbian Medical Association, Belgrade, Serbia; 10 Faculty of Medicine, Institute of Histology and Embryology, University of Belgrade, Belgrade, Serbia

**Keywords:** arterial supply, interpeduncular cisternal segment, measurements, micromorphology, oculomotor nerve

## Abstract

The aim of this study was a detailed examination of the arterial vascularization of the interpeduncular cisternal part (ICP) of a proximal segment of the human oculomotor nerve (ON). The blood vessels of the ONs were carefully microdissected and studied in 30 brain hemispheres using 6.3× to 20× magnification of the stereoscopic microscope. The arteries were injected with the mixture of 10% India ink and gelatin. For better understanding, one brainstem specimen was prepared following a histological procedure, transversely serially cut into 5-μm thick slices, and stained with Luxol fast blue. Another injected midbrain specimen was cut in 1-mm-thick transverse sections, completely cleared with methylsalycilate, and analyzed under transmitted light. The common oculomotor arteries (COAs) and the small oculomotor branches, which participate in the vascularization of the dorsal and ventral surfaces of the ICPs of ONs, were analyzed and their diameters were measured. The dorsal COA was present in 83.33% of the ONs, one per nerve, with an average diameter of 213.57 μm. The ventral COAs were found in 76.67% of the ONs, one per nerve, with an average diameter of 137.64 μm. The mean diameter of the dorsal COAs was significantly higher than the mean diameter of the ventral COAs (*p* < 0.001). The oculomotor fine central vessels supplied the oculomotor root exit zone (OREZ) with the central type of myelin, and distally, fascicles of the rest of ICS with the peripheral type of myelin. The results describing the ON arteries may have diagnostic and microsurgical significance. The lack of detailed morphological analysis of the dorsal region related to the ventral arterial vessels of the ICP, along with measurements of the oculomotor arterial diameters, guided us to conduct this anatomical research to improve diagnostic procedures and the quality of microsurgical interventions in this region.

## Introduction

The oculomotor nerve (ON) is the largest and most complex of the three ocular motor nerves. The nerve fibers of ON are for the somatic motor innervation of superior, inferior, and medial recti, inferior oblique, and levator palpebrae superioris muscles, containing also visceral motor presynaptic parasympathetic axons for the supply of sphincter pupillae and ciliary muscles (through the ciliary ganglion) (Carpenter, 1991; Melling et al., 2003). The axon bundles of the ON (third cranial nerve) emerge from the mesencephalon at the border of the bottom of the interpeduncular fossa, that is at the level of the oblique oculomotor sulcus of the cerebral crus, then join together and quickly form the oculomotor trunk (Standring, 2021). Between the roots of this nerve, and occasionally through the trunk itself, individual branches of the posterior cerebral artery often pass through, especially the thin peduncular branches, but also the strong collicular artery (Milisavljević et al., 1986). Otherwise, all axons of the ON originate from the ipsilateral somatic column, except for the fibers for the superior rectus muscle, which come from the opposite nuclear column of the 3rd nerve (Adams et al., 2008; Vitošević et al., 2013).

The interpeduncular fossa represents a central depressed area between the medial surfaces of cerebral crura, behind and below the mammillary bodies, and rostrally and above the uppermost part of the pons. A relatively small space contains the root axons of the ONs as well as a group of significant blood vessels, the posteromedial central arteries or interpeduncular perforating arteries (IPA). The IPAs are classified in three groups regarding the field of supply, the position, and the origin: (a) thalamoperforating arteries (anterior group, from the posterior cerebral artery, PCA), (b) anterior mesencephalic perforating arteries (middle group, from PCA), and (c) posterior mesencephalic perforating arteries (posterior group, from the basilar artery, BA). The short circumferential branches or peduncular arteries originate from mesencephalic and thalamoperforating arteries. All the aforementioned arteries are closely related and primarily contribute to the vascularization of the cisternal segment of the ON (Marinković et al., 1986; Pedroza et al., 1986; Uz and Tekdemir, 2006; Morales-Roccuzzo et al., 2024).

The first, initial segment of the ON is described as cisternal because it passes through the interpeduncular cistern. The root of the ON courses very deep within the interpeduncular fossa in close contact with a group of interpeduncular arteries. This proper cisternal or interpeduncular part of the ON has rarely been analyzed in the current literature due to its inaccessible location and therefore attracted our attention in the present study. The next part of the cisternal segment of the ON is within the arachnoidal sheath, known as supracavernous or subcavernous, before it enters the cavernous sinus (Iaconetta et al., 2010; Park et al., 2017).

The ON first passes through the interpeduncular cistern, between the posterior cerebral artery (PCA) and the superior cerebellar artery (SCA) (Meybodi et al., 2018). Aneurysms of the PCA or SCA can therefore compress this part of the nerve (Yasargil, 1984; Belotti et al., 2020). The third nerve then extends anteriorly and downward, just below and laterally to the posterior communicating artery (PCoA), and inferomedial to the uncus of the parahippocampal gyrus and the tentorial incisure (notch) (Rhoton, 2000; Park et al., 2017). The close relationship of the ON and the PCoA explains why a lesion of the third nerve is one of the first signs of an aneurysm of this artery (Yasargil, 1984; Belotti et al., 2020). Similarly, the close relationship of the third nerve and the tentorial incisure is the anatomical basis for a lesion of the ON in the case of tentorial herniation of the uncus and parahippocampal gyrus (Cadena et al., 2017).

The ON then enters the dural roof of the cavernous sinus, through the so-called oculomotor triangle, and then continues through the lateral wall of the sinus, just above the trochlear nerve, the ophthalmic nerve, and medially, the abducens nerve (Martins et al., 2006; Takahashi, 2010; Park et al., 2017). These close neural relationships account for the occurrence of complete unilateral ophthalmoplegia, dilated pupils, headache, and upper facial anesthesia in cavernous sinus syndrome (Takahashi, 2010; Rodge et al., 2023). Finally, the ON enters the orbit through the superior orbital fissure at the orbital apex and innervates the corresponding muscles of the eye (Iaconetta et al., 2010).

The aim of this microanatomical research was to examine the neurovascular topographic and morphometric characteristics of arteries supplying the proximal, interpeduncular part of a cisternal segment of the ON. The lack of detailed measurements of oculomotor arterial diameters and comparisons between dorsal and ventral arterial vessels prompted us to propose necessary anatomical support to enhance diagnostic procedures and the quality of microsurgical interventions in this region.

## Materials and methods

For this microanatomical study of injected blood vessels of the ONs, we examined 30 human cerebral hemispheres with no morphological changes, from the collection of the Laboratory for Vascular Morphology. This report exclusively compares the frequencies and diameters of small arteries in specimens of 30 ONs; therefore, age, gender, disease, and other factors were not used to determine from which case the sample was taken. Metric characteristics of oculomotor arterial supply, the origins and diameters of feeding arteries, are also included in this brief research report. We perfused the cerebral arterial system with warm water mixed with a 5% neutral buffered formalin solution, and we finally intra-arterially injected a 10% mixture of India ink and melted gelatin through the basilar and internal carotid arteries. After a minimum period of 4 weeks of fixation, the brain specimens were meticulously dissected. Microdissection of injected blood vessels of 30 human cerebral hemispheres with the oculomotor nerves, using microinstruments, and all measurements were analyzed under the zoom microscope (Leica MZ6), and photographed by a digital photo camera (Leica DFC295). We engaged the specific software (Leica Interactive Measurements) for realizing different kinds of measurements. The vascular network of the ONs and the topographic relationships with the surrounding arteries and veins were drawn in pre-prepared schemes. The data obtained for each specimen were incorporated into the schematic drawings and tables.

For better understanding, the brainstem specimen was fixed in formalin, dehydrated, cleared, and embedded in paraffin. The specimen was transversely serially cut into 5-μm-thick slices and mounted on highly adhesive glass slides. Classic histochemical stain for the visualization of myelin sheath, Luxol fast blue was used to precisely show the position of the cisternal segment of the oculomotor nerve and its central myelin-peripheral myelin transition zone (MTZ). We also used a specimen of the midbrain with arteries filled with India ink and gelatin and prepared 1-mm-thick transverse sections. The sections were completely cleared with methylsalycilate following the procedure of Spalteholz, and the microvessels of the longitudinally sectioned cisternal segment of ON, its root exit zone, and intramesencephalic part were analyzed under transmitted light.

### Statistical analysis

Quantitative experimental data were analyzed using IBM SPSS Statistics version 25.0 software package (SPSS, Inc., Chicago, IL, United States). The statistical analyses comprised descriptive statistics, including frequencies, minimum and maximum values, mean values, and standard deviations of the measured data. The normality of distribution was tested applying the Kolmogorov–Smirnov test, Shapiro–Wilk test and boxplot graphical method of representation. A *t*-test for independent samples was used to compare the means of two groups, diameters of dorsal-related arteries related to the ventral oculomotor arterial vessels. The probability level of *p* < 0.05 was considered a statistically significant difference.

## Results

**Figure 1:**
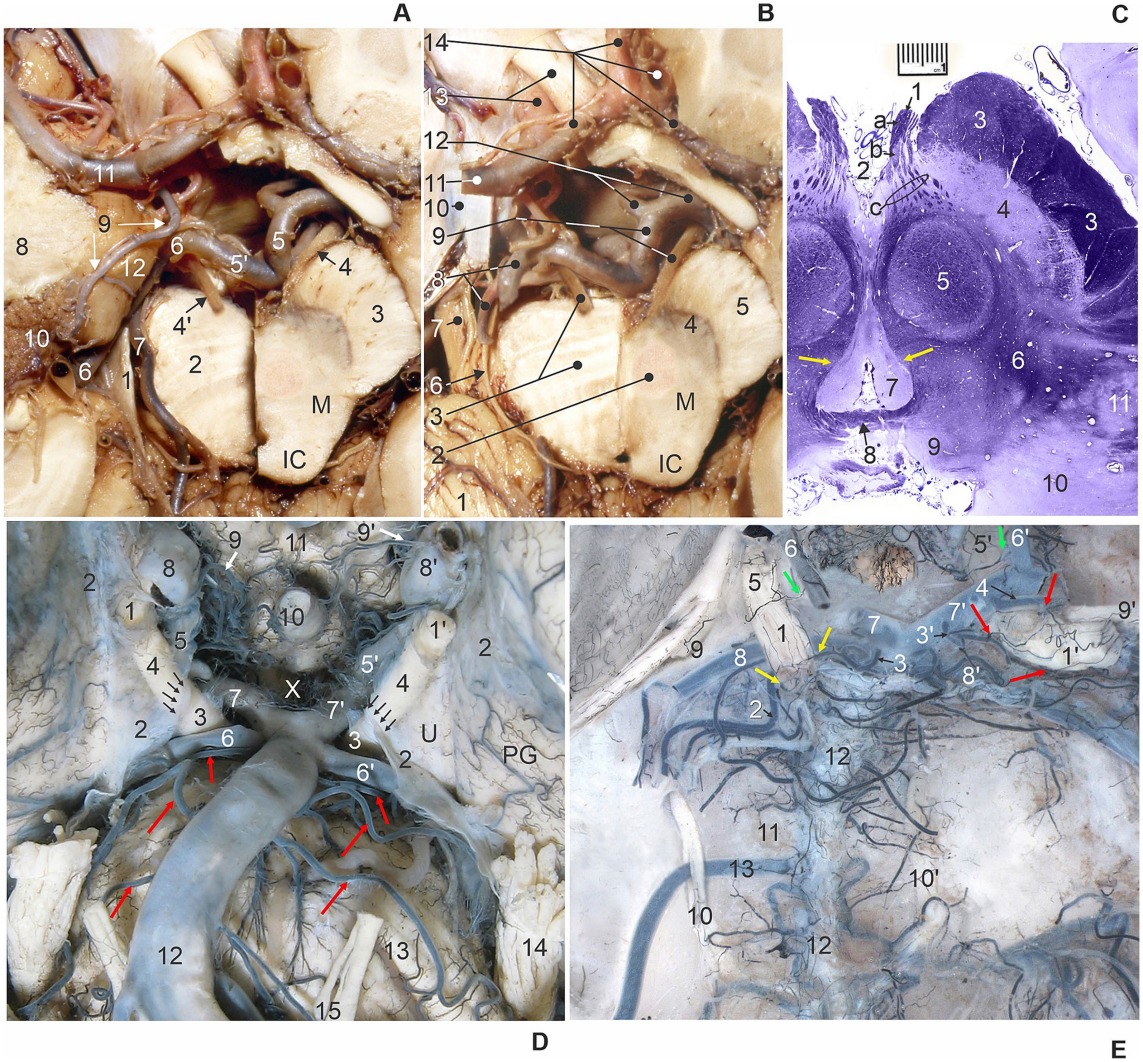
**(A)** Transverse section of the right half of the midbrain (M) at the level of the inferior colliculus (IC). 1, tentorial notch (incisure); 2, left half of pons; 3, right cerebral crus; 4 and 4′, right and left oculomotor nerve (cut); 5 and 5′, right and left PCA – P1 segments; 6, left PCA – P2 segment; 7, left SCA; 8, temporal lobe; 9, left AChA; 10, choroid plexus of lateral ventricle; 11, left middle cerebral artery; 12, uncus (superior view, the skull base, cerebrum and brainstem transected, and brain vessels exposed). **(B)** Transverse section of the right half of the midbrain (M) at the level of the inferior colliculus (IC) from the previous figure after removal of the left temporal lobe. 1, cerebellum; 2, red nucleus; 3, left half of pons and left oculomotor nerve (cut); 4, substantia nigra; 5, cerebral crus; 6, left trochlear nerve; 7, left trigeminal nerve; 8, left posterior cerebral artery (PCA) and superior cerebellar artery (SCA); 9, right PCA – P1 segment and oculomotor nerve; 10, dura mater over cavernous sinus; 11, left middle cerebral artery; 12, right popsterior communicating artery (PCoA) and right PCA – P2 segment; 13, left internal carotid artery (ICA) and optic nerve; 14, left and right anterior cerebral arteries, A1 and A2 segments (superior view, the skull base, brainstem transected, and dissection of brain vessels). **(C)** Transverse oblique stained section of midbrain through the oculomotor nerve (1): a, interpeduncular cisternal peripheral myelin part, b, interpeduncular cisternal central myelin part, c, intramesencephalic part, and yellow arrows – ONC. 2, interpeduncular fossa; 3, cerebral crus; 4, substantia nigra; 5, red nucleus; 6, tegmentum; 7, periaqueductal gray substance; 8, posterior commissure; 9, superior colliculus; 10, pulvinar; 11, medial geniculate body (Luxol fast blue stain). **(D)** Ventral view of the central basal part of the brain with right (1) and left (1′) oculomotor nerves (ONs). Note the arachnoid mater (2) covering the ON, uncus (U), and parahippocampal gyrus (PG), forming a circular constriction of the ONs (arrows) at the level of termination of the interpeduncular cisternal segments of ONs (3) in the interpeduncular cistern (X), and continuation of the precavernous cisternal segment of ONs (4); 5 and 5′, right and left PCoA; 6 and 6′, right and left SCA; 7 and 7′, right and left P1 segments of PCA; 8 and 8′, right and left ICA; 9 and 9′, right and left superior hypophyseal arteries; 10, pituitary stalk; 11, optic chiasm; 12, basilar artery and long pontine arteries (red arrows); 13, pons; 14, left trigeminal nerve; 15, left abducens nerve. **(E)** Dorsal view of the posterior cranial fossa and injected brainstem arteries with pia mater after removal of the brainstem. The left interpeduncular cisternal segment of ON (1) is supplied by the ventral branch (left yellow arrow) from a long pontine artery (2), and the dorsal branch (right yellow arrow) from a diencephalic perforating artery (3). The right interpeduncular cisternal segment of ON (1′) is supplied by the ventral branch (left lower red arrow) and dorsal branch (left upper red arrow) from a thalamoperforating artery (3′), and dorsal branch (right red arrow) from a collicular artery (4). The left and right precavernous cisternal segments of ONs (5 and 5′) receive branches (green arrows) from the left and right PCoAs (6 and 6′); 7 and 7′, left and right P1 segments of PCA; 8 and 8′, left and right SCA; 9 and 9′, left and right trochlear nerves; 10 and 10′, left and right abducens nerves; 11, clivus; 12, basilar artery; 13, left anterior inferior cerebellar artery (AICA) courses between the two roots of left abducens nerve (10).

The first, cisternal intracranial segment of ON extended from its point of exit from the cerebral crus of mesencephalon until the penetration into the lateral dural wall of the cavernous sinus (Figures 1A,B). The rootlets of the ON have left the majority of the medial surface of the mesencephalic crus from the oblique oculomotor sulcus, and formed the cisternal segment of a compact ON trunk inside the interpeduncular cistern, and with the continuation through its lateral subarachnoid extension (Figures 1C,D). Considering pedagogical, clinical, and scientific purposes, we subdivided the cisternal segment of ON into two parts: (a) the interpeduncular or proximal part and (b) the precavernous or distal part.

**Figure 2:**
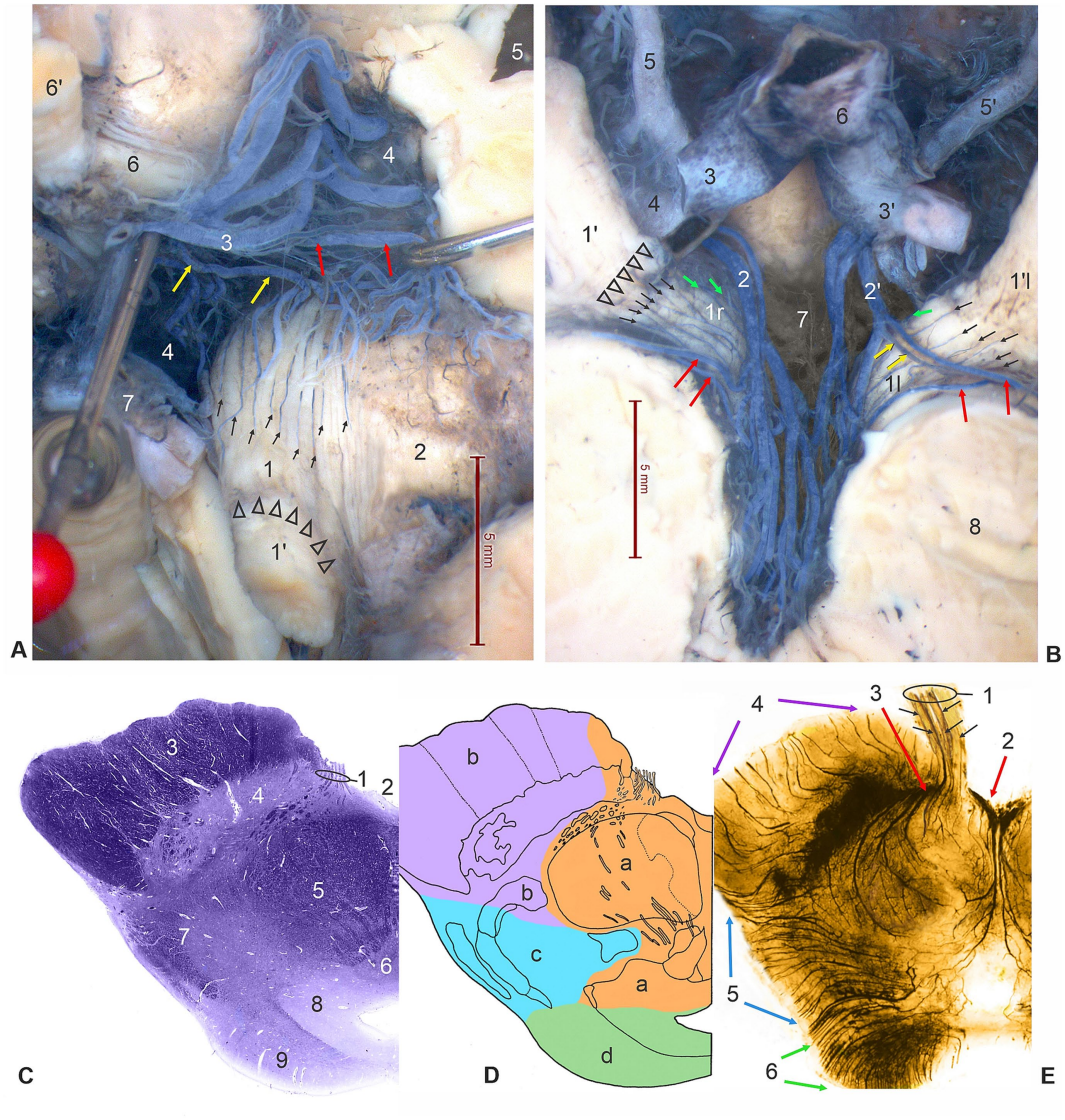
**(A)** Dorsal surface of the left interpeduncular cisternal segment of ON (1) is supplied by slender longitudinal vessels (small arrows) from the common oculomotor artery (yellow arrows) and from a peduncular artery (red arrows) for supply of the left cerebral crus (2), originating from a thalamoperforating artery (3) (elevated by a pin). Arrowheads indicate a circular constriction of the ON at the level of the beginning of the distal part of the cisternal segments of ON (1′). 4, interpeduncular fossa and posterior perforated substance; 5, third ventricle (opened); 6, right interpeduncular, proximal cisternal segment of ON; 6′, right precavernous, distal cisternal segments of ON; 7, P1 segment of PCA (dorsal and right anterior view of ONs, dissection of specimen injected with India ink). **(B)** Ventral surfaces of the right and left interpeduncular, proximal cisternal segments of ONs (1r and 1l), subdivided by constriction (arrowheads) at the place of beginning of precavernous, distal cisternal segments of ON, supplied by fine parallel vessels (small arrows) originating from a peduncular arteries (red arrows), branches of anterior mesencephalic perforating arteries (2 and 2′), or strait from the mentioned arteries (green arrows), and from the left common oculomotor artery (yellow arrows). 3, right PCA – P1 segment (cut); 3′, left PCA – P1 segment; 4, right PCA – P2 segment (cut); 5, right PCoA; 5′, left PCoA; 6, basilar artery, terminal part; 7 – interpeduncular fossa; 8, cerebral crus (ventral view, dissection of specimen injected with India ink). **(C)** Transverse stained section of midbrain through the oculomotor nerve (1). 2, interpeduncular fossa; 3, cerebral crus; 4, substantia nigra; 5, red nucleus; 6, oculomotor nuclear complex; 7, tegmentum; 8, periaqueductal gray substance; 9, superior colliculus (Luxol fast blue stain). **(D)** Schematic drawing of the same transverse section of the midbrain at the level of the superior colliculus. Vascular mesencephalic territories are in different colors: orange (a), anteromedial arteries; pink (b), anterolateral arteries; blue (c), lateral arteries; green (d), posterior arteries. **(E)** Transverse, a 1-mm thick cleared section of midbrain and the longitudinally sectioned interpeduncular cisternal segment of oculomotor nerve and its root exit zone (1) with injected arteries (black India ink and gelatin) showing central branches of fine parallel vessels (small arrows) of oculomotor arteries entering the root zone of the third nerve (Spalteholtz technique). 2, medial mesencephalic and 3, lateral mesencephalic perforating arteries (red arrows); 4, peduncular arteries (pink arrows); 5, lateral mesencephalic arteries (blue arrows); posterior mesencephalic arteries (green arrows); colors of arterial groups correspond to the vascular territories in the previous drawing.

(a) The interpeduncular or proximal part of a cisternal segment of ON emerged from the mesencephalic tissue, and its beginning is described as the oculomotor root exit zone (OREZ). The OREZ consists of oculomotor axons surrounded by central type of myelin produced by oligodendrocytes, and also shows the most complex area of central myelin-peripheral myelin transition zone (MTZ), at the level of the beginning of peripheral type of myelin formed by Schwann cells (Figure 1C).

(b) Precavernous or distal part of a cisternal segment of ON had a characteristic position immediately distal to the cleft formed by two large vessels, PCA and SCA. The ON entered, through the collar-like membranous entrance, into the sleeve formed by the arachnoid mater, within a tubular continuation of the subarachnoid space. The ON was in close contact with the inferomedial surface of the uncus, an anteromedial part of the parahippocampal gyrus (Figures 1A,B). At the level of the third nerve entrance into the narrow subarachnoid continuation of the interpeduncular cistern, the ON showed circular depression on its surface, creating a ring-like narrowing of the nerve (Figures 1D,E, 2A,B).

**Figure 3:**
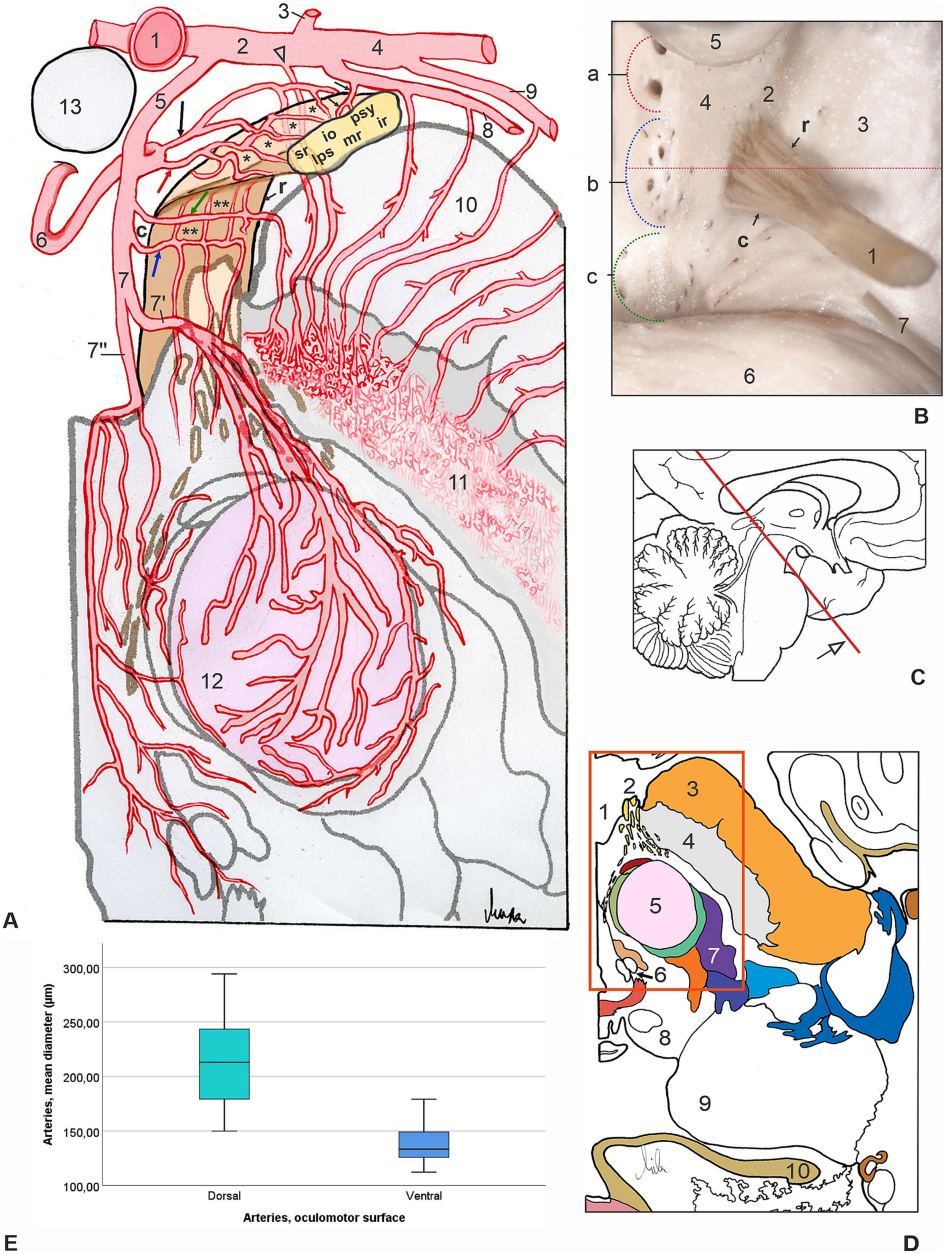
**(A)** Schematic drawing of the most common pattern of arterial supply of a proximal cisternal ON (yellow, reconstruction on section) segment (transverse section, anterior left quadrant of mesencephalon, view from below, see original section on Figure 1B). 1, terminal part of a basilar artery (transected and elevated); 2, P1 segment of left PCA and a tiny peduncular branch (arrowhead); 3, left PCoA; 4, P2 segment of left PCA; 5, interpeduncular perforating artery (IPA); 6, thalamoperforating branch of IPA giving off common oculomotor artery (red arrow) and peduncular artery (black arrow) for the supply of dorsal surface (asterisks) of ON and cerebral peduncle; 7, mesencephalic branch of IPA sending common oculomotor artery (blue arrow) and peduncular artery (green arrow) for the supply of ventral surface (double asterisks) of ON and cerebral peduncle, splitting in lateral mesencephalic perforating artery (7′) and medial (median) mesencephalic perforating artery (7′′); 8, collicular artery giving off peduncular branches and slender oculomotor twig (small arrows); 9, medial posterior choroidal artery; 10, cerebral crus of mesencephalon; 11, substantia nigra and dense capillary plexus; 12, red nucleus; 13, mammillary body. Letters indicate the relative position of axons within the ON, rostral (r) and caudal (c) parts, for the innervation of sphincter pupillae and ciliary muscles (psy), inferior rectus (ir), medial rectus (mr), levator palpebrae superioris (lps), superior rectus (sr), and inferior oblique (io) muscles. **(B)** Ventral view of the proximal cisternal segment of ON (1), rostral (r) and caudal (c) parts, with rootlets emerging from oculomotor sulcus (2) of the cerebral crus of mesencephalon (3). Left half of interpeduncular fossa (4) shows three groups of entrance openings for: (a) thalamoperforating arteries, (b) anterior mesencephalic perforating arteries, and (c) posterior mesencephalic perforating arteries; 5, mammillary body; 6, pons (the red dotted line indicates the plane of a transverse section through the middle of the oculomotor root exit zone and the mesencephalon presented in a previous drawing). **(C)** Midsagittal section of a left cerebral hemisphere, the red line indicates the plane of an oblique cross-section of the brainstem presented in a previous drawing. **(D)** Schematic presentation of the same transverse section of midbrain (see original section on Figure 1B). The red quadrant represents the area shown in the previous drawing. 1, interpeduncular fossa; 2, ON; 3, cerebral crus; 4, substantia nigra; 5, red nucleus; 6, oculomotor nuclear complex; 7, medial lemniscus; 8, superior colliculus; 9, pulvinar; 10, fornix. **(E)** Comparison of the mean diameters (μm) of common oculomotor arteries (COAs) of two surfaces of 30 ONs: dorsal (green) and ventral (blue). *t*-test for independent samples showed that the mean diameter of the dorsal COAs was significantly higher than the diameter of the ventral COAs (*t* = 8.72, *p* < 0.001).

Analysis of arterial vessels for the interpeduncular part of a cisternal segment of ON required removal of the brainstem up to the level of the pontomesencephalic sulcus, cutting and elevating also the terminal part of the basilar artery, firstly accessing the dorsal side of the ON, and secondly the ventral side of the ON (Figures 2A,B, 3A,B). The thalamoperforating branches represent the anterior group of IPA for the supply of the ventromedial part of the thalamus, the caudal part of the hypothalamus, the medial part of the cerebral crus, and ON (Figures 2A, 3A). The anterior mesencephalic perforating arteries, the middle group of IPA, subdivided into medial mesencephalic and lateral mesencephalic perforating arteries, known as anteromedial arteries of the midbrain, for the vascularization of paramedian and central parts of the mesencephalon, the medial part of the cerebral crus, and ON (Figures 2B–E, 3A).

### Arteries of the dorsal surface of the interpeduncular cisternal part of the oculomotor nerve

We have identified two groups of arteries that participate in the vascularization of the interpeduncular cisternal part (ICP) of the ONs: the common oculomotor trunk or oculomotor artery and the small, independent, individual oculomotor branches. A common oculomotor artery (COA) was present in 25 (83.33%) of the dorsal sides of the ONs, always one per nerve, with an average diameter of 213.57 ± 40.42 μm (range 149.82–294.10 μm) (Figures 2A, 3A; Table 1). The origin of the dorsal COA was most often from the thalamoperforating artery of the P1 segment of PCA, in 11 (44%) roots of the ONs, also from the anterior mesencephalic artery in 8 (32%) of the cases, as well as from the collicular artery. The collicular artery, present in all 30 hemispheres, in 26 (86.67%) cases originated from the P1 segment of the PCA, and in 6 (24.00%) cases gave rise to the COA (Table 1). The small individual oculomotor branches existed in all studied ONs, form 2–9, with an average of 3.43 per nerve. More numerous in cases when the COAs were absent, in 5 (16.67%) of the ONs, their number was 8–9 (mean 8.8) (Figures 1E, 2A, 3A-D; Table 1).

**Table 1:** Arteries for the supply of interpeduncular, proximal cisternal segments of 30 ONs.

Oculomotor arteries (OAs) of 30 ONs	OAs, frequency (%), and number per nerve (mean)	OAs, origin, and frequency (%)	OAs, diameter (μm), and min–max (*M* ± SD)
Dorsal surfaces of 30 ONs	Common oculomotor artery 25 (83.33) One per nerve	Thalamoperforating arteries 11 (44.00)	149.82–294.10 (213.57 ± 40.42)
		Anterior mesencephalic 8 (32.00)	
		Collicular artery 6 (24.00)	
	Oculomotor branches 30 (100.00) 2–9 (3.43)	Thalamoperforating, anterior mesencephalic, collicular, peduncular arteries	69.88–119.82 (93.22 ± 12.94)
Ventral surfaces of 30 ONs	Common oculomotor artery 23 (76.67) One per nerve	Anterior mesencephalic 18 (78.26)	112.13–179.11 (137.64 ± 15.53)
		Peduncular artery 5 (21.74)	
	Oculomotor branches 30 (100.00) 3–8 (4.34)	Anterior mesencephalic, peduncular arteries, long pontine arteries	41.64–106.51 (80.87 ± 12.37)

OAs, oculomotor arteries; ONs, oculomotor nerves; M, mean; SD, standard deviation.

OAs were injected with a mixture of India ink and melted gelatin, microdissected under the zoom microscope and the diameters measured using the Leica Interactive Measurements software.

### Arteries of the ventral surface of the interpeduncular cisternal part of the oculomotor nerve

The ventral surface of the ICP of ON was supplied by slender longitudinal vessels from the common oculomotor trunk or artery and/or from small, independent individual branches. A common oculomotor artery (COA) with an average diameter of 137.64 ± 15.53 μm (112.13–179.11 μm) was present in 23 (76.67%) of the ventral surfaces of the ONs, always one per nerve (Figures 2B, 3A; Table 1). The ventral COA most often originated from the anterior mesencephalic artery of the P1 segment of PCA, in 18 (78.26%) of the ONs, and also from the peduncular artery in 5 (21.74%) of the cases. The small individual oculomotor branches were present in all studied ONs, from 3–8, with an average of 4.34 per nerve. In ONs when the ventral COAs were absent, in 7 (23.33%) of the cases, the number of tiny vessels was 6–8 (mean 7.29) (Figures 2B, 3A-D; Table 1).

Comparison of the mean diameters (μm) of COAs for the supply of dorsal and ventral surfaces of ONs applying *t*-test for independent samples showed that the mean diameter of the dorsal COAs was significantly higher than the diameter of the ventral COAs (*t* = 8.72, df = 31.44, *p* < 0.001) (Figure 3E).

## Discussion

The oculomotor nucleus complex in each half of the mesencephalon is composed of the lateral somatic cell column of the oculomotor nucleus, made up of alpha-motor neurons whose axons form intramesencephalic bundles that have the appropriate arrangement. Thus, the fascicles of the inferior oblique muscle are located most laterally; more medially, there are axons for superior rectus muscle; the next group of fibers innervates the medial rectus muscle; and the most medial (therefore, near the midline) innervates the inferior rectus muscle. The caudal central subnucleus contains alpha-motor neurons whose axons form bundles that innervate both the left and the right levator palpebrae superioris muscles and pass together with completely crossed bundles to the superior rectus muscle. In humans, the parasympathetic axons of a group of neurons located above the nuclear complex and the Edinger–Westphal nucleus, pass close to the midline, together with the motor bundles for the inferior rectus muscle (Horn et al., 2009; Vitošević et al., 2013; Konofaou et al., 2019). In a rostrocaudal direction the most rostral position is occupied by axons of the ON for the inferior rectus muscle and the small intraocular muscles receiving parasympathetic fibers, intermediate part contains fibers for the medial rectus and inferior oblique muscles, and the caudal position is for the fibers innervating levator palpebrae superioris and the superior rectus muscles (Vitošević et al., 2013; Park et al., 2017; Standring, 2021). It is accepted but not yet specified that extramesencephalic fibers of ON in men are organized in a topographic way with the appropriate position of the superior and inferior group of axons before they split anatomically in the superior branch (for the levator palpebrae superioris and superior rectus muscles) and inferior branch (for the remaining innervated muscles). The axons for the innervation of the medial rectus muscle occupied the ventral portion of the ON (Atasever et al., 1992). Preganglionic parasympathetic axons belonging to the cisternal segment of ON correspond to the dorsomedial surface of the nerve and are placed superficially (Brazis, 1991; Srinivasan et al., 2015).

The first, cisternal segment of the ON is subdivided into two parts regarding the position and topographic relations: (a) initial part (proper cisternal part), and (b) supracavernous or subcavernous (within the arachnoidal sheath), before it enters the cavernous sinus, then through the superior orbital fissure, and finally inside the orbit (Marinković and Gibo, 1994; Uz and Tekdemir, 2006; Iaconetta et al., 2010; Park et al., 2017). We labeled the first part as the interpeduncular or proximal part of a cisternal segment of ON according to the position inside the interpeduncular fossa of the midbrain. The very specific structure of this unique part of ON is composed of two different kinds of myelin sheets: proximal or central and distal or peripheral myelin. Two groups of glial myelin-producing cells form the myelin of the interpeduncular part of the ON axons: oligodendrocytes that ensheathe up to 40–50 axon segments in the central part of the nerve, and Schwann cells that create the peripheral part of the myelin sheath of only 1 mm of one axon per cell (Standring, 2021). Changes in myelin homeostasis and neurodegeneration are directly associated with reduced brain perfusion. The extension of mesencephalic white matter, or the central myelin area of ON, formed by oligodendrocytes, is particularly sensitive to reduced blood flow and poor oxygenation, with the loss of cells in response (Bouhrara et al., 2020). Astrocytes in the central and fibroblasts in the peripheral parts of the nervous tissue of the interpeduncular part of the ON are different supporting cells that make this part very sensitive to changes in normal arterial supply, and they react to the presence of external mechanical pressure. In cases where an active defensive response is required, reactive astrocytes together with oligodendroglial cells contribute to nerve regeneration after injury, having the main influence in the maintenance of tissue homeostasis (Verkhratsky et al., 2023). Following compression and hypoxia-induced stimulation in the peripheral nervous system, the interactionamong macrophages, fibroblasts, and Schwann cells promotes axon regeneration (Dun and Parkinson, 2020). The OREZ and the myelin transitional zone (MTZ), a week area of the ICP of the ON, are exposed to the possible vascular compression and a disturbance of the intraneural circulation. The length of the oculomotor root exit zone (OREZ), central myelin portion of the interpeduncular part of ON, reported in a previous study varied from an average value of 0.33 mm on the lateral side to 0.5 mm on the medial side (Alfieri et al., 2012). According to another group of authors, the OREZ was longer and had a mean value of 2.75 mm (Quanchareonsap et al., 2023).

The second, intracavernous segment of the ON then enters the dural roof of the cavernous sinus, through the so-called oculomotor triangle, and then continues through the lateral wall of the sinus, just above the trochlear nerve, the ophthalmic nerve, and medially, the abducens nerve (Martins et al., 2006; Takahashi, 2010; Park et al., 2017). These close neural relationships explain the occurrence of complete unilateral ophthalmoplegia, dilated pupils, headache, and upper facial anesthesia in cavernous sinus syndrome (Takahashi, 2010; Rodge et al., 2023). Finally, the ON enters the orbit through the superior orbital fissure at the orbital apex and innervates the corresponding muscles of the eye (Iaconetta et al., 2010).

The limited space of the interpeduncular fossa contains the ONs and numerous vessels of different sizes and positions. The close relationships of the ONs and mentioned vessels were discussed more or less in the previous scientific reports (Yasargil, 1984; Marinković et al., 1986; Milisavljević et al., 1986; Pedroza et al., 1986; Marinković and Gibo, 1994; Cahill et al., 1996; Uz and Tekdemir, 2006; Takahashi, 2010; Esmer et al., 2011; Standring, 2021; Morales-Roccuzzo et al., 2024). Our original classification of the arteries supplying the interpeduncular part of the ON into two groups was based on a detailed study of 30 hemispheres and 30 ONs. Based on the examined material, we concluded that there were two main types of arterial vessels: the common oculomotor artery (COA) or trunk and a single oculomotor branch. The oculomotor branches mainly originate from a larger common stem (COA), always one for a dorsal (in 83.33% of nerves), and one for a ventral (in 76.67% of nerves) surface, but also as independent branches with a different origin. Precise neurovascular micromorphology is essential for practitioners in neurosurgery, neurology, and neuroradiology to deepen their understanding of basic vascular relations. Aneurysms of the BA bifurcation, P1 or P2a segments of the PCA, fused with the ICP of ON, or loop like shape of the PCoA with a vascular compression of the ON (Yasargil, 1984; Takahashi, 2010; Belotti et al., 2020), or neurovascular conflict of the ON by the PCA (Liang et al., 2009; Inoue et al., 2012; Tan et al., 2014; Jo et al., 2015) (or tumors of surrounding structures) represent a small segment of numerous reports of abnormal arterial compression on the ICP of ON.

For the first time regarding the arterial supply of the interpeduncular cisternal parts (ICPs) of the oculomotor nerves (ONs), we examined their dorsal and ventral surfaces, compared the origins of feeding vessels as well as their calibers. We studied large thalamoperforating arteries and their branches, including dorsal COAs and peduncular arteries, which, together with the collicular artery, were mostly in close contact with the dorsomedial surface of the cisternal segment of ON. The ventrolateral surface of ON was related to the cerebral crus of the mesencephalon and received branches of the anterior mesencephalic, peduncular, ventral COAs, and long pontine arteries. Our investigation proved that the mean diameter of the COAs of the dorsal surfaces of the ONs was larger than the mean diameter of the ventral COAs, statistically significant at *p* < 0.001. Larger dorsal oculomotor arteries are dominant in the supply of ICP of Ons, and we hypothesize that neurovascular compression and ischemia of the dorsal side of the ICP of ON may cause more serious lesions followed by ON palsy.

## Data Availability

The raw data supporting the conclusions of this article will be made available by the authors, without undue reservation.
